# How agency shapes behavior and performance: the triple impact of control-feedback on stimulus–response learning, motor reinforcement, and motivated action selection

**DOI:** 10.3389/fpsyg.2026.1730025

**Published:** 2026-01-28

**Authors:** Noam Karsh

**Affiliations:** 1 Social and Motor Cognition Lab, Department of Psychology, Tel-Hai College, Kiryat Shmona, Israel; 2 Special Population Advance Research and Clinical Center (SPARC), University of Haifa, Haifa, Israel

**Keywords:** action selection, agency, control, motivation, motor performance, reinforcement, stimulus–response association

## Abstract

**Introduction:**

Agency confirmation via control feedback (e.g., an immediate sensory consequence of one’s action) has been shown to motivate action choice and reinforce motor responses. Recent work also demonstrated that it qualitatively improves motor performance. The present study tested the hypothesis that this improvement arises because control feedback selectively strengthens stimulus–response (S–R) associations, and further examined its reinforcing impact on motor responses and action choice to provide an integrated account of how agency confirmation shapes behavior and performance.

**Methods:**

Three experiments employed an acquisition-test paradigm. During acquisition, specific stimulus–response combinations triggered an immediate perceptual effect, while other combinations produced no effect (Experiments 1 and 3) or a delayed effect (Experiment 2). In the test phase, the perceptual effect depended solely on the response (Experiments 1 and 2) or was absent (Experiment 3). Experiment 3 also included a free-choice phase assessing the motivating impact of control feedback on voluntary action selection and explicit knowledge regarding the S–R pairings.

**Results:**

Control feedback enhanced S–R learning, yielding faster and more accurate performance for previously reinforced pairings compared to delayed or no-effect conditions. Immediate response-contingent effect independently facilitated motor execution (Experiments 1 and 2), and reinforced S–R pairings biased action choice preference (Experiment 3) even without explicit awareness of the pairings.

**Discussion:**

Agency confirmation via control feedback exerts a triple and partially dissociable influence on behavior, enhancing S–R learning, reinforcing motor execution, and motivating voluntary action. The findings inform models of action control and motor skill learning.

## 1 Introduction

Early psychological theories (White, 1959; DeCharms, 1968; Skinner, 1953; Higgins, 2012) emphasized the motivating and functional value of control exercised through one’s own actions. Empirical evidence confirmed this premise (Eitam et al., 2013; Karsh and Eitam, 2015a; Reis et al., 2023; see also Stephens, 1934), highlighting the role of agency confirmation following an action-effect as an independent motivator for action. Specifically, studies have shown that the mere sensory effect following a response, the most fundamental form of control feedback, reinforces both the speed and frequency of actions in and of itself (Eitam et al., 2013; Karsh and Eitam, 2015a; Karsh et al., 2016; Karsh et al., 2020; Karsh et al., 2021; Penton et al., 2018; Tanaka et al., 2021; Wen and Haggard, 2018; Ren et al., 2023; Hemed et al., 2022; Karsh et al., 2025).

The Control-Based Response Selection (CBRS) framework (Karsh and Eitam, 2015b) integrates these findings, proposing that agency confirmation, whether via sensorimotor prediction or cognitive inference of self-causality (e.g., “I did it”), carries intrinsic reward value. According to this framework, these two routes to establishing agency have differential impacts on motivated voluntary action selection and motor reinforcement.

Notably, extensions of this work have demonstrated qualitative contributions of control feedback to motor performance, extending beyond response times and frequency (Karsh et al., 2024b; Karsh et al., 2024c; Karsh et al., 2024a). The authors proposed that a qualitative contribution to motor performance may stem from the control-feedback reinforcing the association between the relevant stimulus and response in a selective manner, a mechanism that received support in a previous work (Tanaka et al., 2021). However, this proposition has not yet been directly empirically tested.

The evaluation of agency, the causal link between one’s own action and its perceptual effect, relies on confirming sensorimotor predictions (Blakemore et al., 1999; Frith et al., 2000; Synofzik et al., 2008) and non-motor expectations (Wegner et al., 2004; Moore et al., 2009). The comparator model (Miall and Wolpert, 1996; Wolpert and Flanagan, 2001; Welniarz et al., 2021) provides a computational framework for motor-based agency evaluation, proposing that forward sensory predictions, derived from an efference copy of the motor command, are compared with incoming sensory feedback to detect spatial and temporal discrepancies (e.g., prediction errors). Minimal temporal and spatial discrepancies between predicted and actual sensory feedback confirm sensorimotor predictions, contributing to the perceived causal link between one’s action and effect (e.g., the sense of agency; Synofzik et al., 2008; Wen, 2019; Frith et al., 2000). At a conceptual level, agency evaluation may also involve cognitive inference or self-causality attribution, based on the alignment of anticipated and perceived action outcomes alongside contextual cues (Wegner et al., 2004; Moore et al., 2009; Aarts et al., 2005).

Confirmation of agency through temporally contiguous and spatially predicted action effects has been shown to independently reinforce response selection. Pioneering work by Eitam et al. (2013) demonstrated that an immediate perceptual effect following an action increases response speed, even when feedback about success in task performance (e.g., a running score) is provided. Karsh and Eitam (2015a) further established that an action’s perceptual effect reinforces response frequency, even when the effect is irrelevant to the task, and this action tendency undermines task performance.

Notably, different levels of response selection (e.g., deciding “what to do” versus “how to do it”) exhibit varying sensitivities to different types of control-relevant information (Karsh and Eitam, 2015a). For example, in speeded reaction-time tasks, the facilitating effect of action-effect feedback on response times is more sensitive to agency-relevant physical parameters, such as temporal contiguity and spatial predictability, and less influenced by explicit control knowledge (Karsh and Eitam, 2015a), the probability of an effect occurring without a motor action (Hemed et al., 2022), or conceptual outcome-oriented goals like maximizing monetary rewards (Karsh et al., 2020). This suggests that the facilitating effect on response times can emerge directly through pre-conceptual reinforcement of motor execution through confirmed sensorimotor predictions. In contrast, response frequency in a free-choice action-selection task (which may reflect a voluntary decision of “what to do”) is more sensitive to explicit control knowledge (Karsh and Eitam, 2015a) and action-effect contingency (Hemed et al., 2022). Thus, such a higher-level stage of action-selection is likely modulated to a greater extent by conceptual processes, including explicit control knowledge, self-agency attribution, and general control beliefs (Ren et al., 2023), which can also modulate general attentional engagement and response readiness (Karsh et al., 2016, 2021; Ren et al., 2023).

An extension of this work demonstrated a qualitative contribution of control feedback to motor control performance (Karsh et al., 2024b; Karsh et al., 2024c; Karsh et al., 2024a). In a study by Karsh et al. (2024c), Go/No-Go (GNGT) and Stop-Signal (SST) tasks were used to assess automatic and controlled inhibition, respectively (Verbruggen and Logan, 2008; Schachar et al., 2007; Raud et al., 2020). The study showed that an immediate action-effect (a brief white flash following a Go response) increased response speed in Go trials and the likelihood of successful No-Go trials in the GNGT, without affecting the stop-signal reaction time (SSRT) in the SST. The authors proposed that in the GNGT, the immediate action-effect enhances the selective association between the Go stimulus and the Go response, speeding up Go trial responses and reducing the likelihood of a Go response to irrelevant stimuli (e.g., the No-Go stimulus) without requiring additional cognitive control resources. In the SST, where response inhibition cannot rely on automatic stimulus–response associations due to the stop signal following the Go signal on the same trial, an immediate action effect did not modulate SSRT.

Another study (Karsh et al., 2024b) examined the impact of action effects on action control in individuals with and without ADHD, using a task where participants responded only to rare colored stimulus sequences. An own-action effect (immediate vs. 400 ms delayed action-effect) consistently reduced false alarms in both groups and improved practice efficiency. These findings can be explained by the contribution of control-feedback to the development of a representational structure that links perception and action in a selective manner.

A previous work by Tanaka et al. (2021) supports this mechanism by demonstrating that when control feedback (e.g., immediate vs. delayed action effects) was contingent on specific stimulus–response (S–R) combinations, responses were faster in S–R trials that triggered an immediate effect. Their findings underscore the stimulus’s role in the impact of action-effect on response times, which may indicate that an action’s perceptual effect could strengthen the selective association between a stimulus and its corresponding motor representation. However, their study provides only indirect support for enhanced selective S–R association by control feedback for several reasons. First, immediate action effects were not restricted to correct responses, such that control feedback was not selectively contingent on specific S–R pairings. Second, the single-phase design does not permit firm conclusions regarding whether selective S–R associations were formed and maintained once control feedback was no longer present, beyond local facilitation effects. Finally, although immediate effects reliably shortened response times, they were not consistently accompanied by improvements in accuracy, and in some cases, were associated with increased errors, arguing against a qualitative enhancement of performance driven by control feedback.

The present study was designed to address these issues and directly test the hypothesis that control feedback enhances selective S–R associations, playing a crucial role in motor performance. In addition, to advance an integrated account of how agency shapes motivated behavior and performance, I independently assessed reinforcement from response-contingent control feedback and the motivating impact of control feedback on voluntary action selection, as proposed by the CBRS framework (Karsh and Eitam, 2015b; see also Hemed et al., 2022).

### 1.1 The current study

I conducted three experiments using an adapted version of Eitam et al.’s (2013) speeded reaction-time task (Experiments 1 and 2) and the free-choice variant of Karsh and Eitam (2015a) in Experiment 3. Unlike Eitam et al. (2013) and Tanaka et al. (2021), the task consisted of two consecutive phases, an acquisition phase and a test phase. In acquisition, control feedback (e.g., an immediate, brief white flash of the circle) was contingent on a specific combination of stimulus (color) and response (key). In the subsequent test phase, the perceptual effect was determined solely by the response, irrespective of stimulus color. Notably, neither the perceptual effect nor the color was task-relevant as participants were instructed to respond as fast and accurately as possible based on the arrow direction. Across all experiments, I investigated whether S–R pairings that trigger control feedback during acquisition improve performance on the same S–R combinations in the test phase. In Experiment 1, I compared immediate-effect trials to no-effect trials. Experiment 2 replicated Experiment 1 while comparing immediate-effect trials to 600 ms delayed-effect trials, a delay range previously shown to impair implicit agency measures (Haggard et al., 2002; Blakemore et al., 1999), including reinforcement from control feedback (Tanaka et al., 2021; Wen, 2019; Eitam et al., 2013). Experiment 3 further replicated the findings of Experiment 1 in a free-choice acquisition phase suitable for measuring the impact of control feedback on voluntary action selection (Karsh and Eitam, 2015a; see also Hemed et al., 2022).

The study employed a within-subject design, allowing me to test whether S–R combinations that produced control feedback during acquisition improved test-phase performance specifically on compatible trials, in which the same S–R pairing had previously triggered an immediate effect, relative to incompatible trials, in which the S–R pairing had not. Second, I assessed the immediate impact of control feedback on motor reinforcement (Experiments 1–2), which I expected to be reflected in shorter response times on trials where an immediate effect was contingent on the response in the test phase. Third, the impact of control feedback on motivated action choice was expected to be reflected in a biased selection of responses that generated an immediate action effect in the free-choice acquisition phase (Experiment 3). Finally, I explored whether participants’ explicit knowledge of S–R pairings that trigger control feedback moderated its effect on performance (Experiment 3).

## 2 Experiment 1: enhanced stimulus–response association following immediate compared to no-effect trials

### 2.1 Methods

#### 2.1.1 Participants

Twenty-four participants (19 females; Age: *M* = 24.87, *SD* = 3.45) completed the study at the University of Haifa. *A priori* power analysis was conducted to target the interaction effect in the two-way repeated-measures ANOVA, which corresponds to the study design. Using GPower (Faul et al., 2007) with a medium effect size (*f* = 0.25), four repeated measurements, and *α* = 0.05, the analysis indicated that a sample size of *N* = 24 provides 80% power. All participants reported normal or corrected-to-normal vision and normal color vision. Each received course credit or a 40 NIS (~$11) voucher. The study was approved by the Tel-Hai College IRB (06–11-24).

#### 2.1.2 Stimuli and procedure

Participants sat in a quiet room facing a computer monitor and a standard PC keyboard. They placed their right and left index fingers on the designated “K” (right) and “D” (left) keys, respectively. On each trial, a red or blue circle (diameter = 1.5 cm) containing a left or right pointing arrow appeared at the upper-center of the game window (5 cm*10 cm) and rapidly descended for 1.1 s to the bottom of the game window. Participants pressed the key matching the arrow as quickly as possible. The inter-trial interval was set to 0.1 s, and the trial length was independent of response time.

**Figure 1:**
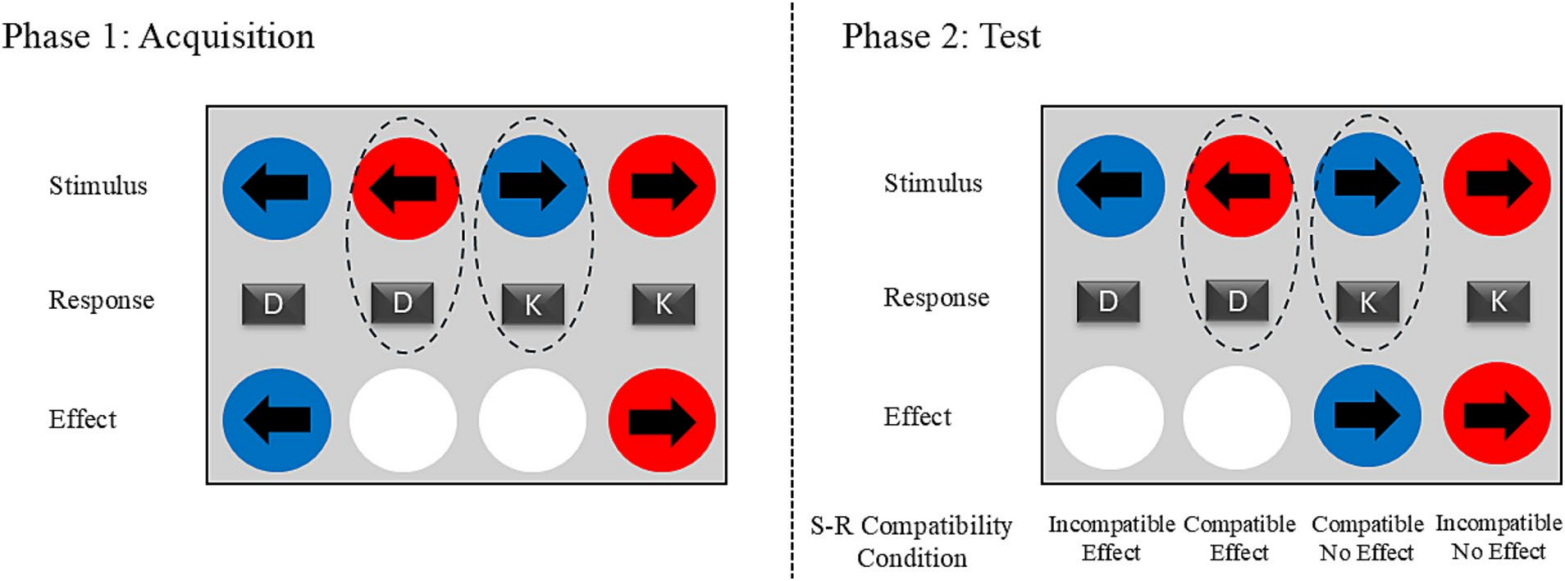
An illustration of trial logic for a sample combination of stimulus (color)–response (key) mapping. During the acquisition phase, the perceptual effect (indicated by a white flash) is associated with specific stimulus–response pairs. In the test phase, it depended only on the response that was made. Thus, each condition in the test phase was defined by whether the trial was compatible with the color–response combination that produced an immediate effect during acquisition (compatible vs. incompatible) and whether it had a response-contingent effect (effect vs. no effect) during the test.

The task consisted of 400 trials in two consecutive phases, with participants unaware of the phase transition (Figure 1). In the acquisition phase (200 trials), a specific color–response pairing determined whether a correct response produced an effect. In effect trials, a correct response triggered an immediate 100 ms white flash that replaced the circle’s color; in no-effect trials, the circle continued to descend until the trial ended. Each participant was randomly assigned to one of two color–response mappings that produced the effect (e.g., left-key after red and right-key after blue, or vice versa). Color and effect were task-irrelevant as participants’ goal was to match their response to the arrow direction.

In the test phase (200 trials), effect occurrence depended solely on the response key (e.g., left for “D” or right for “K” or vice versa), which was counterbalanced across participants. This design tested both the immediate impact of response-contingent effects and transfer from S–R regularities acquired in the first phase.

### 2.2 Results

The same filtration procedure was applied across all three experiments. Correct trials with RT < = 200 ms or > = 800 ms were excluded (~0.1%). I then removed trials that deviated by more than 2 SD in each direction from the condition’s mean RT (5% of the remaining trials) separately for the acquisition and test phases. Incorrect trials were excluded from RT analyses. Two-tailed *p*-values are reported for all *t*-tests unless otherwise specified.

#### 2.2.1 Acquisition

Mean RTs did not differ between pairings that triggered an effect (*M* = 509, *SD* = 29) and those that did not (*M* = 508, *SD* = 30), *t*(23) = −0.52, *p* = 0.60, *dz* = 0.1, 95% CI [−5, 3]. Accuracy was lower for S–R pairings triggering an immediate effect (*M* = 0.86, *SD* = 0.06) than for S–R pairings that did not (*M* = 0.88, *SD* = 0.07), *t*(23) = 2.41, *p* = 0.024, *dz* = 0.49, 95% CI [0.003, 0.04].

#### 2.2.2 Test

**Figure 2:**
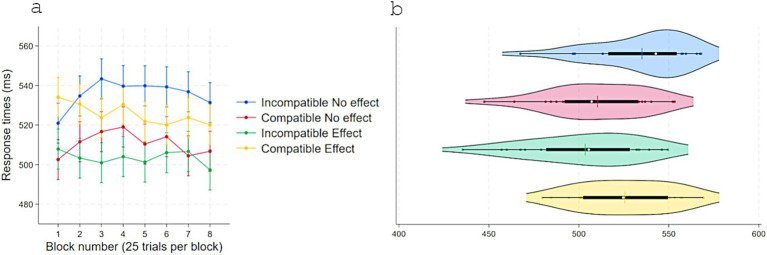
Response times (ms) in experiment 1 as a function of S–R compatibility and response-contingent effect. **(a)** Marginal means across eight test blocks (25 trials each) with error bars depicting 95% confidence intervals. **(b)** Violin plots showing individual data points, with a color-matched vertical line indicating the mean of each condition, a white dot marking the median, and a horizontal black bar depicting the interquartile range.

A two-way repeated-measures ANOVA on RTs with Response-contingent effect (Effect vs. No-effect) and Compatibility (Compatible vs. Incompatible) with acquisition pairings delivering an effect showed a main effect of Response-contingent effect, *F*(1, 23) = 12.63, *p* = 0.001, η^2^partial = 0.35, with faster responses on Effect trials (*M* = 512, *SD* = 29) than No-effect trials (*M* = 518, *SD* = 30). Critically, there was a strong interaction effect, *F*(1, 23) = 43.97, *p* < 0.001, η^2^partial = 0.65. No other effects approached statistical significance (Figure 2). Tukey-adjusted comparisons indicated that, in No-effect trials, RTs were shorter for Compatible (*M* = 510, *SD* = 27) than Incompatible pairings (*M* = 535, *SD* = 26), Contrast = −24, *p* < 0.001, 95% CI [−38, −10]. In Incompatible trials, RTs were shorter with an Effect (*M* = 503, *SD* = 30) than with No-effect, Contrast = −31, *p* < 0.001, 95% CI [−45, −17]. When both compatibility and response-contingent effect were present, responses were slower (*M* = 525, *SD* = 25) than when only one of these factors was present, i.e., No-effect-Compatible, Contrast = 15, *p* = 0.027, 95% CI [1, 29], and Effect-Incompatible, Contrast = 22, *p* = 0.001, 95% CI [8, 35].

**Figure 3:**
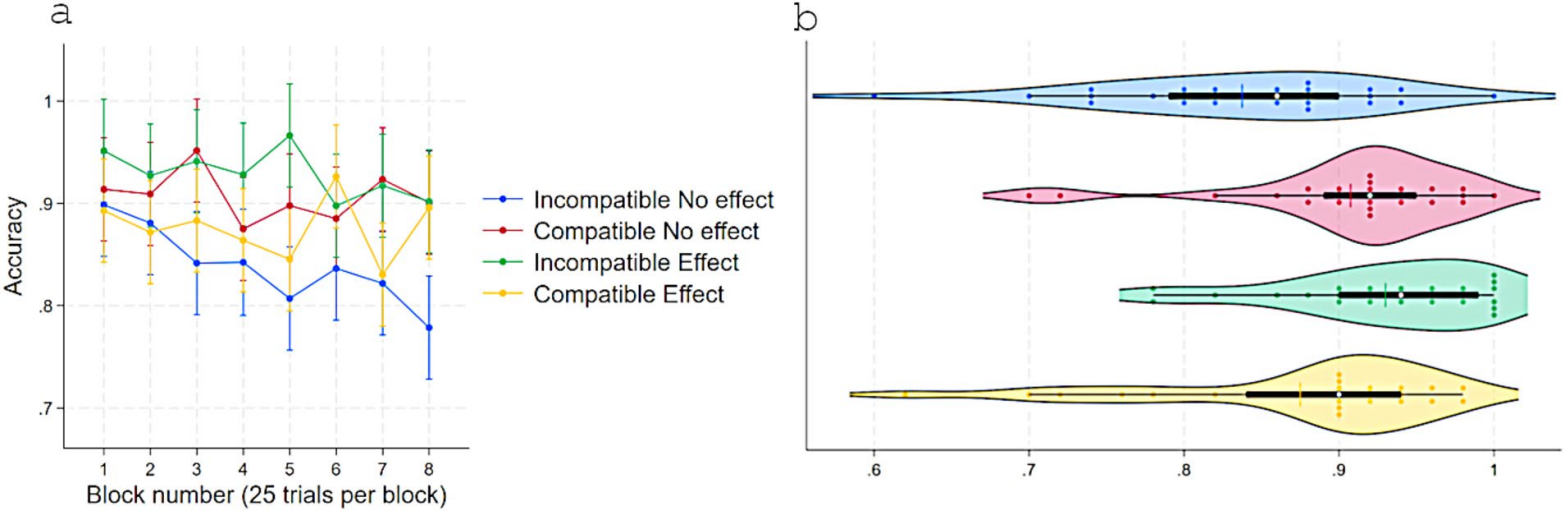
Accuracy in experiment 1 as a function of S–R compatibility and response-contingent effect. **(a)** Marginal means across eight test blocks (25 trials each) with error bars depicting 95% confidence intervals. **(b)** Violin plots showing individual data points, with a color-matched vertical line indicating the mean of each condition, a white dot marking the median, and a horizontal black bar depicting the interquartile range.

A two-way repeated measure ANOVA on Accuracy showed a parallel pattern (Figure 3). A main effect of Response-contingent effect, *F*(1, 23) = 6.9, *p* = 0.015, η^2^partial = 0.23 (Effect: *M* = 0.89, *SD* = 0.08; No-effect: *M* = 0.87, *SD* = 0.08) and a robust interaction with Compatibility, *F*(1, 23) = 27.99, *p* < 0.001, η^2^partial = 0.54. No other terms were significant. Further Tukey-adjusted comparisons revealed that in No-effect trials, accuracy was higher for Compatible (*M* = 0.90, *SD* = 0.07) than Incompatible (*M* = 0.83, *SD* = 0.09) pairings, Contrast = −0.07, *p* = 0.002, 95% CI [0.02, 0.11]. In Incompatible trials, accuracy was higher with Effect (*M* = 0.93, *SD* = 0.06) than No-effect, Contrast = 0.09, *p* < 0.001, 95% CI [0.04, 0.13]. When trials were both Compatible and with a response-contingent effect, accuracy (*M* = 0.87, *SD* = 0.09) did not differ from No-effect-Compatible trials, Contrast = −0.03, *p* = 0.23, 95% CI [−0.07, 0.01], and was lower than Effect-Incompatible trials, Contrast = −0.05, *p* = 0.016, 95% CI [−0.10, −0.008].

These findings provide direct evidence that control-feedback (an immediate action-effect) enhances the formation of S–R associations that triggered the perceptual effects and, in turn, selectively improves motor performance as indicated by higher accuracy and shorter RT for the relevant S-R combinations. In addition, the findings clarify that the immediate reinforcing impact of the response-contingent effect on motor execution dissociates from its role in S–R learning, indicating distinct processes.

## 3 Experiment 2: enhanced stimulus–response association following an immediate compared to a delayed effect

Experiment 2 was designed to replicate the findings of Experiment 1 and to more directly connect the influence of action-effects to theoretical models of agency determination. The same experimental procedure was used, but instead of comparing immediate effect trials with No-effect trials, the comparison was between immediate effect and 600 ms Delayed effect trials. Previous research has shown that such delays impair implicit measures of agency (Haggard et al., 2002; Blakemore et al., 1999; Wen, 2019) and reduce reinforcement from control feedback (Tanaka et al., 2021; Eitam et al., 2013).

### 3.1 Methods

#### 3.1.1 Participants

Twenty-four participants (18 females; Age: *M* = 25.16 years, *SD* = 2.65) completed the experiment at the University of Haifa. Sample-size planning followed Experiment 1. All reported normal or corrected-to-normal vision and normal color vision, and received the same compensation as in Experiment 1.

#### 3.1.2 Stimuli and procedure

The task and apparatus were identical to Experiment 1, with one modification. Instead of comparing immediate and no-effect trials, I replaced the no-effect condition with a 600 ms delayed effect condition. Accordingly, the circle descent duration was also extended to 1.7 s. This manipulation preserves the physical stimulation of the perceptual effect across conditions while disrupting temporal contiguity, which robustly impairs implicit agency measures.

### 3.2 Results

The same filtration procedure was applied. Correct trials with RT < = 200 ms or > = 800 ms were excluded (~0.6%). I then removed trials that deviated by more than 2 SD in each direction from the condition’s mean RT, separately for the acquisition and test phases (~4.8% of the remaining trials).

#### 3.2.1 Acquisition

RTs did not differ between S–R pairings producing immediate (*M* = 557, *SD* = 36) versus delayed (*M* = 554, *SD* = 35) effects, *t*(23) = −1.52, *p* = 0.14, *dz* = 0.31, 95% CI [−7, 1]. Likewise, accuracy did not differ between Delayed (*M* = 0.89, *SD* = 0.07) and Immediate (*M* = 0.90, *SD* = 0.07) effect trials *t*(23) = −0.4, *p* = 0.69, *dz* = 0.08, 95% CI [−0.02, 0.01].

#### 3.2.2 Test

**Figure 4:**
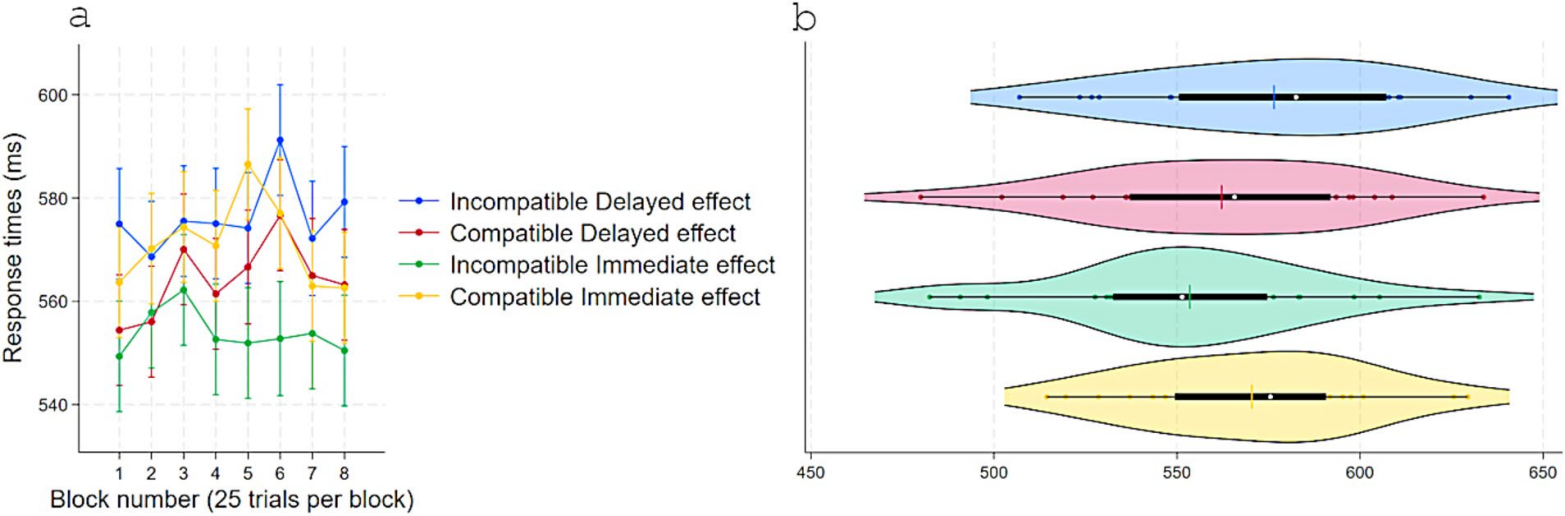
Response times (ms) in Experiment 2 as a function of S–R compatibility and response-contingent effect. **(a)** Marginal means across eight test blocks (25 trials each) with error bars depicting 95% confidence intervals. **(b)** Violin plots showing individual data points, with a color-matched vertical line indicating the mean of each condition, a white dot marking the median, and a horizontal black bar depicting the interquartile range.

A two-way repeated-measure ANOVA on RT with Response-contingent effect (Immediate vs. Delayed) and Compatibility (Compatible vs. Incompatible) as a within-subject factors revealed a main effect of Response-contingent effect, *F*(1, 23) = 5.87, *p* = 0.023, η^2^partial = 0.20, with faster RTs in Immediate (*M* = 560, *SD* = 34) than Delayed (*M* = 564, *SD* = 36) trials, and a substantial interaction, *F*(1, 23) = 22.00, *p* < 0.001, η^2^partial = 0.48. No other effects approached statistical significance (Figure 4). Tukey-adjusted comparisons showed that, in Delayed trials, RTs were shorter for Compatible (*M* = 562, *SD* = 36) than Incompatible (*M* = 576, *SD* = 35) pairings, Contrast = −14, *p* = 0.028, 95% CI [−27, −1]. In Incompatible trials, RTs were shorter for Immediate (*M* = 553, *SD* = 35) than Delayed, Contrast = −22, *p* < 0.001, 95% CI [−36, −9] effect trials. When trials were both Compatible and with Immediate effect, RTs (*M* = 570, *SD* = 30) did not differ from Compatible-Delayed trials (Contrast = 8.26, *p* = 0.32, 95% CI [−4, 21]) and were longer than Incompatible-Immediate trials (Contrast = 16, *p* = 0.008, 95% CI [3, 30]).

**Figure 5:**
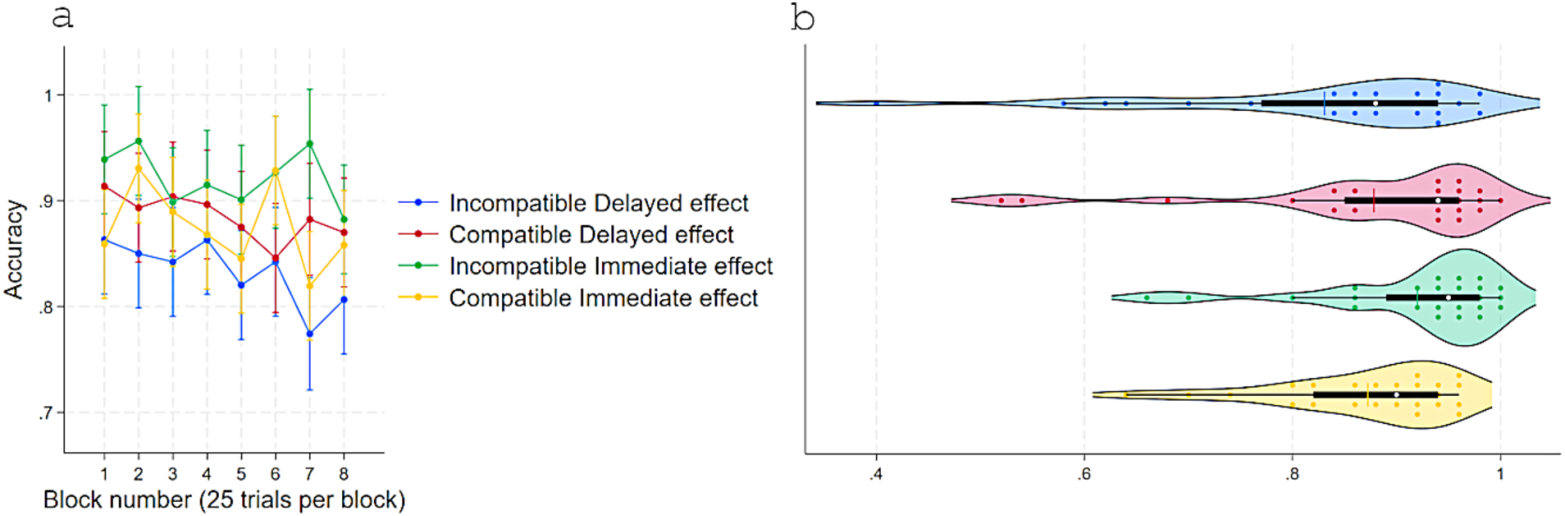
Accuracy in experiment 1 as a function of S–R compatibility and response-contingent effect. **(a)** Marginal means across eight test blocks (25 trials each) with error bars depicting 95% confidence intervals. **(b)** Violin plots showing individual data points, with a color-matched vertical line indicating the mean of each condition, a white dot marking the median, and a horizontal black bar depicting the interquartile range.

Accuracy analyses mirrored the RT pattern (Figure 5). The two-way repeated-measures ANOVA showed a main effect of Response-contingent effect, *F*(1, 23) = 7.85, *p* = 0.01, η^2^partial = 0.25 (Immediate: *M* = 0.89, *SD* = 0.08; Delayed: *M* = 0.86, *SD* = 0.12) and a strong interaction effect with Compatibility, *F*(1, 23) = 20.88, *p* < 0.001, η^2^partial = 0.47. Other terms were not significant. Further Tukey-adjusted comparisons showed that in Delayed effect trials, accuracy was higher for Compatible (*M* = 0.87, *SD* = 0.12) than Incompatible (*M* = 0.83, *SD* = 0.14) pairings, Contrast = 0.04, *p* = 0.018, 95% CI [0.006, 0.08]. In Incompatible trials, accuracy was higher for Immediate (*M* = 0.92, *SD* = 0.09) than Delayed effects, Contrast = 0.08, *p* < 0.001, 95% CI [0.04, 0.12]. When trials were both Compatible and with Immediate effect, accuracy (*M* = 0.87, *SD* = 0.08) did not differ from Compatible-Delayed (Contrast = −0.005, *p* = 0.97, 95% CI [−0.04, 0.03]) and was lower than Incompatible-Immediate trials (Contrast = −0.04, *p* = 0.018, 95% CI [−0.08, −0.006]).

Experiment 2 fully replicated Experiment 1 and linked the selective S–R learning benefit to the temporal contiguity of action-effects, a crucial parameter for evaluating self-agency. Also consistent with Experiment 1, the control-feedback enhancement of S–R learning dissociated from the immediate reinforcing impact of response-contingent immediate effect on motor responses.

## 4 Experiment 3: motivated action choice and explicit knowledge of S–R pairings

Experiment 3 was conducted to replicate and extend the findings of Experiments 1 and 2 within a free-choice paradigm during the acquisition phase. This design is particularly suited to capture the motivating influence of control-feedback on action selection. Because such motivational processes are sensitive to explicit control knowledge and contextual cues relevant to agency attribution, I expected S–R pairings that produced an immediate effect to bias participants’ action choices. In this way, Experiment 3 allowed examining whether S–R associations formed via action-effects can emerge spontaneously in a free-choice context and be strengthened by the motivating impact of control-feedback. This mechanism would, in turn, facilitate learning through repeated exposure to S–R regularities that provide control feedback. In addition, I assessed participants’ explicit knowledge of the specific stimulus (color) and response (key) combinations that triggered action-effects and examined the contribution of such control explicit knowledge to action selection and subsequent performance.

### 4.1 Methods

#### 4.1.1 Participants

Fifty-five students (38 females; Age: *M* = 24.10, *SD* = 2.78) participated in the study. To test whether control-driven action choices during acquisition predict performance in compatible S–R pairings during test, *N* = 55 provided 80% power to detect a medium effect in linear multiple regression. One participant reporting abnormal color vision was replaced. The other participants reported having normal or corrected-to-normal vision and received course credit or a 40 NIS voucher.

#### 4.1.2 Stimuli and procedure

The task was based on Experiment 1 with key changes in the acquisition phase. The arrows inside the circle were removed, and participants were instructed to choose one of the two response keys randomly and spontaneously on each trial, avoiding pre-planning. As in Experiment 1, immediate effects followed specific S–R combinations while the remaining combinations produced no effect. The test phase was identical to Experiment 1 except that no responses produced an effect, allowing to attribute any differences in performance between conditions solely to S–R associations established during acquisition.

### 4.2 Results

The same filtration procedure was applied. Trials with RT < = 200 ms or > = 800 ms were excluded (~4%). I then removed trials that deviated by more than 2 SD in each direction from the condition’s mean RT, separately for the acquisition and test phases (~4% of the remaining trials).

#### 4.2.1 Acquisition

The proportion of effect-delivering responses (*M* = 0.68, *SD* = 0.22) exceeded the 50% chance level, *t*(54) = 6.13, *p* < 0.001, *d*z = 0.82, 95% CI [0.62, 0.74], although such biased action choice interferes with their task performance of responding randomly. In addition, RTs were shorter in trials where the S–R pairing did not deliver an effect (*M* = 407, *SD* = 75) than when it did (*M* = 417 ms, *SD* = 75), *t*(54) = −2.80, *p* = 0.005, *dz* = 0.39, 95% CI [−17, −3], which may plausibly stem from enhanced control processes for learning action-effect contextual regularities in effect trials.

#### 4.2.2 Test

**Figure 6:**
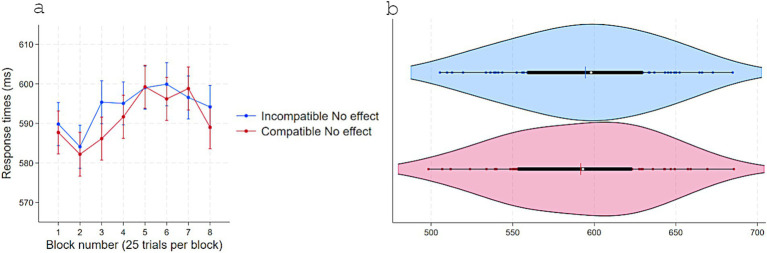
Response times (ms) in Experiment 3 as a function of S–R compatibility: **(a)** Marginal means across eight test blocks (25 trials each) with error bars depicting 95% confidence intervals and **(b)** violin plots showing individual data points, with a color-matched vertical line indicating the mean of each condition, a white dot marking the median, and a horizontal black bar depicting the interquartile range.

**Figure 7:**
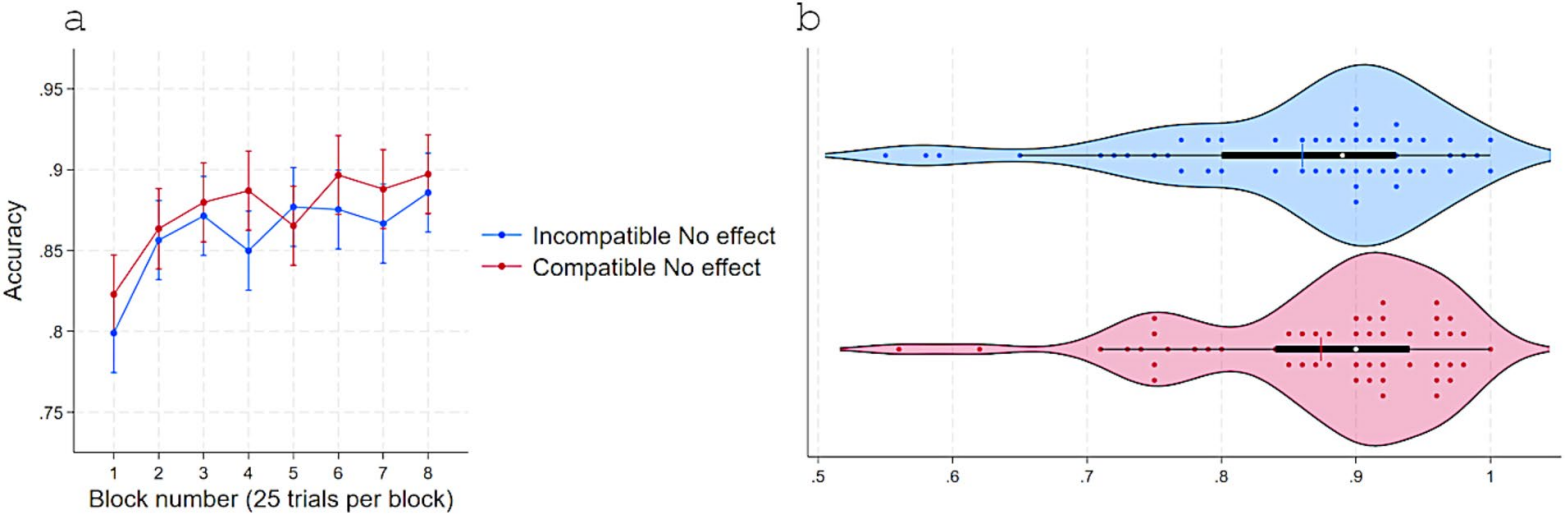
Accuracy in Experiment 3 as a function of S–R compatibility. **(a)** Marginal means across eight test blocks (25 trials each) with error bars depicting 95% confidence intervals. **(b)** Violin plots showing individual data points, with a color-matched vertical line indicating the mean of each condition, a white dot marking the median, and a horizontal black bar depicting the interquartile range.

Replicating Experiments 1 and 2, RTs were shorter when responses were compatible (*M* = 591, *SD* = 45) versus incompatible (*M* = 594, *SD* = 44) with S–R pairings that delivered immediate effects in acquisition, *t*(54) = 2.22, *p* = 0.03, *dz* = 0.30, 95% CI [0.28, 5] even though no effect appeared in the test phase following a response (Figure 6). Consistently, accuracy was higher for Compatible (*M* = 0.87, *SD* = 0.09) than Incompatible (*M* = 0.86, *SD* = 0.10) pairings, *t*(54) = −2.12, *p* = 0.038, *dz* = 0.28, 95% CI [−0.02, −0.0007] (Figure 7).

#### 4.2.3 Action-choice bias and test performance

I further tested whether a higher proportion of effect-consistent choices during acquisition predicted a larger compatibility advantage. To this end, I conducted linear multiple regression analyses predicting RT and accuracy separately, with the proportion of effect trials entered as a continuous predictor, along with S–R compatibility and their interaction. The analyses revealed no effects on either accuracy or RT. I then conducted *post hoc* analysis predicting response times and accuracy separately, additionally including Block number (8 test blocks of 25 trials each) as a continuous predictor and its interactions with S-R compatibility and the proportion of effect trials. The model predicting accuracy [*F*(7, 54) = 2.89, *p* = 0.012, *R*^2^ = 0.02] reveals a two-way interaction between S-R compatibility and the proportion of effect trials (coefficient = 0.15, *p* = 0.03, 95% CI [0.01, 0.29]). In addition, a three-way interaction was found between compatibility, the proportion of effect trials, and Block number (coefficient = −0.02, *p* = 0.035, 95% CI [−0.04, −0.001]). I then conducted simple slopes analyses to evaluate the contribution of the proportion of effect trials to accuracy in the first test block for each S-R compatibility condition, where I expected the largest transfer of S-R associations to occur. No significant trend was observed for either Compatible (dy/dx = 0.09, *p* = 0.2, 95% CI [−0.05, 0.24]) or Incompatible (dy/dx = −0.03, *p* = 0.69, 95% CI [−0.19, 0.12]) trials.

#### 4.2.4 Explicit control knowledge

A post-experiment electronic survey item, asking participants to mark possible color and key combinations that produced an immediate effect during the acquisition phase, showed that only 54.55% (30 participants) correctly identified both correct color–key combinations. Consistently, those with accurate explicit control knowledge (*M* = 0.78, *SD* = 0.21) showed a higher proportion of effect trials during acquisition than those without (*M* = 0.56, *SD* = 0.17), *t*(53) = −4.02, *p* < 0.001, *d* = 1.10, 95% CI [−0.32, −0.10]. Importantly, both groups, with [*t*(29) = 7.15, *p* < 0.001, *d* = 1.30, 95% CI [0.70, 0.86]] and without [one-tailed *t*(24) = 1.96, *p* = 0.03, *d* = 0.39, 95% CI [0.49, 0.63]] explicit control knowledge, nevertheless selected effect-producing responses above chance.

Finally, a mixed-model ANOVA with Control explicit knowledge (with vs. without) as a between-subjects factor and S–R compatibility as a within-subjects factor on RTs showed only the main effect of Compatibility but no interaction, *F*(1, 53) = 0.12, *p* = 0.73, η^2^partial = 0.002. Similarly, the same analysis conducted on accuracy showed only the main effect of Compatibility but no interaction, *F*(1, 53) = 0.06, *p* = 0.813, η^2^partial = 0.001. Thus, while explicit knowledge was associated with (but not required for) a tendency to select effect-producing responses consistent with motivation from the control account, the performance benefit of compatible S–R pairings in the test phase was not moderated by explicit control knowledge.

## 5 Discussion

The present study aimed to directly examine how agency confirmation via control feedback (e.g., temporally contiguous action–effects versus no effect or lagged effects) functions as a crucial factor for learning (S-R associations), with direct implications for improving motor performance. Across three experiments using an acquisition–test task, I investigated whether control feedback selectively strengthens S–R associations and thereby improves performance when those S–R pairs are reencountered. To provide an integrative account of how control feedback shapes behavior, I further examined its reinforcing impact on motor execution when control feedback was contingent on the response (Experiments 1 and 2), and its motivating impact on voluntary action selection (Experiment 3).

Three main findings emerged. First, S–R pairs that produced immediate effects during acquisition yielded selective performance benefits in the test phase when such immediate effects were no longer provided. The performance advantage was reflected in faster and more accurate responses for compatible than incompatible S–R combinations, delivering either no effect (Experiment 1) or a 600 ms delayed effect (Experiment 2) in the acquisition phase. Second, during the test phase, immediate response-contingent effects facilitated motor performance, especially when the same S–R pair had not previously produced control feedback (Experiments 1–2). Third, in a free-choice acquisition phase (Experiment 3), participants preferentially chose effect-producing responses governed by S–R rules, and this bias was independent of explicit knowledge about the underlying S–R rules.

Together, the results support a triple impact of control feedback on learning (S–R association), motor reinforcement, and motivated action selection. These influences appear to arise from two partially independent routes, a preconceptual motor route that reinforces execution through confirmation of sensorimotor predictions, and a context-dependent route that enhances selective S–R associations and biases voluntary action choice (Eitam et al., 2013; Hemed et al., 2022; Karsh and Eitam, 2015a, Karsh and Eitam, 2015b; Karsh et al., 2016, 2020; Tanaka et al., 2021).

### 5.1 Control feedback strengthens S–R associations and improves performance

The primary theoretical contribution of this work is the demonstration that agency confirmation through control feedback selectively strengthens S–R associations, thereby enhancing subsequent performance on those same S–R combinations. Prior research indicated that immediate (vs. delayed) effects speed responses when such effects are tied to specific stimuli (Tanaka et al., 2021). Our two-phase design formally separated acquisition from test, revealing a transfer benefit in motor performance that tracked which S-R pairs had previously produced control feedback, when control feedback is no longer provided.

Unlike Tanaka et al. (2021), the advantage in the present study’s acquisition-test paradigm was evident in both response time and accuracy, indicating a learning-dependent change in S-R mapping strength that qualitatively contributes to motor performance beyond mere response facilitation. Importantly, our conclusions diverge from Tanaka et al. (2021), who attribute the facilitation produced by control feedback solely to strengthened S-R associations. In contrast, our data support the view that control feedback also influences action choice and motor reinforcement, presumably via two partially dissociable mechanisms.

Within the Control-Based Response Selection (CBRS) framework (Karsh and Eitam, 2015b; Hemed et al., 2022), these findings correspond to a higher-level route in which cognitive inferences of self-causality take into account contextual regularities, enhancing the associative link between a stimulus and its response. However, although the S–R rule was accessible and could be intentionally monitored, the data show that this strengthening does not depend on explicit knowledge of control. Namely, roughly half of the participants failed to report the correct S–R pairings that provided control feedback, yet both knowledge groups displayed similar compatibility advantages in the test phase. In this sense, the process through which S-R associations are reinforced by control feedback can be analogous to reinforcement-learning processes (Glimcher, 2011; O’Doherty et al., 2015), with control feedback functioning as an intrinsic reward that can operate independently of explicit awareness of agency.

These results also converge with broader theories of event coding and ideomotor learning, which posit that perception and action are integrated through shared representational codes that link sensory to motor components of an event (Hommel et al., 2001; Tanaka et al., 2021). From this perspective, control feedback may act as a binding cue that promotes the development of event files, uniting stimulus and response codes into a coherent representation. Thus, the present data extend ideomotor principles by demonstrating that agency confirmation may play a vital role in forming these S–R associations.

The findings can also inform us about the nature of the acquisition and retrieval processes of such learning. The absence of facilitation and even poorer performance in immediate-effect trials during acquisition suggests that learning S-R pairings predicting control feedback may recruit top-down control and attentional processes (Frings et al., 2020). Only after repeated exposure with immediate action-effects do S-R associations become automatized and capable of enhancing observed performance when retrieved (Logan, 1988; Hommel, 2004).

Critically, the performance benefit driven by automatized S–R associations emerged only in trials without an immediate response-contingent effect in the test phase (the No-effect and Delayed-effect trials). This pattern was not predicted *a priori* in Experiment 1 but was replicated in Experiment 2. The absence of a compatibility benefit in Compatible–Immediate Effect trials may reflect interference arising from the previously learned S–R–Immediate Effect rule and the newly introduced R–Immediate Effect rule during the test phase. This change in control-relevant (e.g., immediate action-effect) regularities likely necessitates relearning, engaging top-down control processes that can inhibit or override the automatic retrieval of established S–R associations. Importantly, this form of interference is absent in incompatible trials, where the S–R pairings did not produce immediate effects during acquisition. In these trials, the immediate reinforcing impact of response-contingent effects in the test phase becomes evident. Consistently, in No-effect and Delayed-effect trials, which do not impose a change in the control-relevant rule, previously acquired S–R associations can be retrieved automatically without interference, thereby facilitating performance in compatible trials.

### 5.2 Dissociable impacts on S–R learning and motor reinforcement

Immediate response-contingent effects produced clear motor facilitation relative to no-effect (Experiment 1) and delayed-effect (Experiment 2) conditions, replicating prior demonstrations that confirmation of sensorimotor predictions reinforces motor execution (Eitam et al., 2013; Karsh et al., 2016, 2020). The observed dissociation between motor facilitation driven by immediate response-contingent effects and S–R transfer driven by prior feedback history supports the notion that motor reinforcement from a response-contingent control feedback and enhanced performance from S-R associative learning rely on partially independent control-relevant computations (Hemed et al., 2022; Karsh and Eitam, 2015b). Moreover, when control feedback was determined by S–R combinations in the acquisition phase (Experiments 1 and 2), no response facilitation was observed in effect trials. This is consistent with the view that immediate reinforcement of motor execution arises from response-contingent (not S–R contingent) effects, critical for confirming sensorimotor predictions (whereas non-motor cognitive mechanisms track contextual regularities that strengthen specific S–R associations).

### 5.3 The interplay between motivated action selection and S–R learning

In Experiment 3, participants were biased to select responses that produced effects more frequently, even though instructed to choose randomly. This replicates the motivating impact of control feedback on voluntary action selection (Karsh and Eitam, 2015a) and extends it by showing that this motivation is informed by contextual S–R regularities. Consistent with previous work (Karsh and Eitam, 2015a), the preference was stronger among participants with accurate explicit knowledge but was also evident without it, suggesting that conceptual knowledge can amplify rather than be necessary for the motivating impact of control on action choice. Related evidence shows that stronger perceived agency increases action readiness and reduces inhibitory control (Ren et al., 2023). Such divergence encourages future research to elucidate the conditions under which agency confirmation may increase action readiness and challenge inhibitory control efforts, versus conditions where it enhances the S–R association that contributes to the automatic inhibition of inappropriate responses (e.g., Karsh et al., 2024b; Karsh et al., 2024c).

Our data suggest, albeit inconclusively, that repeated control-seeking during acquisition can feed back into learning, producing performance advantages. The absence of robust effects on accuracy and RT likely reflects the relatively large number of action–effect trials for most participants, which offered sufficient exposure that may have reduced between-subject differences in S–R learning. Future work should employ a design that allows a more precise characterization of how S–R associations decay over time. For example, increasing the number of alternative response options (e.g., from two to four) would yield greater between-subject variability in exposure to control feedback during acquisition, enabling a more sensitive examination of how such variability predicts the persistence or decline of compatibility effects throughout the test phase.

Taken together, the current findings converge with previous work indicating a qualitative contribution of agency to cognitive and perceptual processing (Wen et al., 2018; Kumar et al., 2015; Hon and Yeo, 2021). For example, studies of attentional processing show that sensory events perceived as consequences of one’s own actions receive priority in attentional processing, resulting in more efficient selection than physically identical but non-agentic events (Kumar et al., 2015). Complementary work further demonstrates that a sense of agency over an outcome selectively enhances episodic memory for the associated stimulus, suggesting that agency may act as a distinctive self-related cue that supports later retrieval (Hon and Yeo, 2021).

Focusing on motor performance, the present study demonstrates that agency confirmation through control feedback shapes behavior through multiple and partly independent routes. Control feedback not only reinforces motor execution and motivates action selection but also acts as a cue for forming and strengthening S–R associations. When a perceptual effect reliably follows a specific S–R pairing, the cognitive system treats this regularity as a marker of self-generated influence, strengthening the perception–action mapping.

From a developmental and applied perspective, these mechanisms may underline early motor learning in infancy, where agency confirmation via control feedback guides actions and further improves motor skills (Watanabe and Taga, 2006; Hauf et al., 2004; Ravid-Roth et al., 2025). In rehabilitation and motor-training contexts, designing tasks with control feedback can be an efficient strategy to enhance patients’ sense of control and motor performance by strengthening S–R associations.

## Data Availability

The datasets presented in this study can be found in online repositories. The names of the repository/repositories and accession number(s) can be found at: Open Science Framework (OSF): https://osf.io/j2seb/overview?view_only=f98ccc46f1be477f8c0fda0ea5faa42e.
